# Genome Sequencing Reveals a Mixed Picture of SARS-CoV-2 Variant of Concern Circulation in Eastern Uttar Pradesh, India

**DOI:** 10.3389/fmed.2021.781287

**Published:** 2022-01-07

**Authors:** Hirawati Deval, Dimpal A. Nyayanit, Shailendra Kumar Mishra, Pragya D. Yadav, Kamran Zaman, Prem Shankar, Brij R. Misra, Sthita Pragnya Behera, Niraj Kumar, Abhinendra Kumar, Pooja Bhardwaj, Gaurav Raj Dwivedi, Rajeev Singh, Anita M. Shete, Priyanka Pandit, Ashok K. Pandey, Girijesh Kumar Yadav, Shashi Gupta, Manoj Kumar, Asif Kavathekar, Ravi Shankar Singh, Sanjay Prajapati, Rajni Kant

**Affiliations:** ^1^Indian Council of Medical Research (ICMR)-Regional Medical Research Centre, Gorakhpur, India; ^2^Indian Council of Medical Research (ICMR)-National Institute of Virology, Pune, India; ^3^All India Institute of Medical Sciences, Gorakhpur, India

**Keywords:** SARS-CoV-2, variant of concern, whole-genome sequencing, mutations, Eastern Uttar Pradesh India, COVID-19 breakthrough infection

## Abstract

Uttar Pradesh is the densely populated state of India and is the sixth highest COVID-19 affected state with 22,904 deaths recorded on November 12, 2021. Whole-genome sequencing (WGS) is being used as a potential approach to investigate genomic evolution of the severe acute respiratory syndrome coronavirus 2 (SARS-CoV-2) virus. In this study, a total of 87 SARS-CoV-2 genomes−49 genomes from the first wave (March 2020 to February 2021) and 38 genomes from the second wave (March 2021 to July 2021) from Eastern Uttar Pradesh (E-UP) were sequenced and analyzed to understand its evolutionary pattern and variants against publicaly available sequences. The complete genome analysis of SARS-CoV-2 during the first wave in E-UP largely reported transmission of G, GR, and GH clades with specific mutations. In contrast, variants of concerns (VOCs) such as Delta (71.0%) followed by Delta AY.1 (21.05%) and Kappa (7.9%) lineages belong to G clade with prominent signature amino acids were introduced in the second wave. Signature substitution at positions S:L452R, S:P681R, and S:D614G were commonly detected in the Delta, Delta AY.1, and Kappa variants whereas S:T19R and S:T478K were confined to Delta and Delta AY.1 variants only. Vaccine breakthrough infections showed unique mutational changes at position S:D574Y in the case of the Delta variant, whereas position S:T95 was conserved among Kappa variants compared to the Wuhan isolate. During the transition from the first to second waves, a shift in the predominant clade from GH to G clade was observed. The identified spike protein mutations in the SARS-CoV-2 genome could be used as the potential target for vaccine and drug development to combat the effects of the COVID-19 disease.

## Introduction

The newly identified severe acute respiratory syndrome coronavirus 2 (SARS-CoV-2), discovered in Wuhan in December 2019 received global attention due to its extensive and rapid transmission and infectivity (1). The virus spread over 200 countries within 5 months through international travels and its airborne transmission via respiratory droplets and contact routes. The first case of COVID-19 in India was reported from Kerala state on January 30, 2020 (2). As on November 12, 2021, over 34 million SARS-CoV-2 cases with 4.6 lakh deaths were reported in India (3).

During the second wave of SARS-CoV-2, the emergence of the highly infectious and/or virulent SARS-COV-2 variant of concern (VOC) has triggered intensive genomic surveillance. According to the global initiative on sharing all influenza data (GISAID) nomenclature system, the SARS-CoV-2 variants were distributed into currently eight major clades including from early split L (belongs to the Wuhan reference strain) and S, to the further evolution of L into V and G, and later of G into GH, GR, and GV, and more recently GR into GRY (4). Recent studies demonstrated the identification of key spike mutations, insertions/deletions in the SARS-CoV-2 genome via genome sequencing (5). Hence, the whole genome sequence (WGS) analysis has become an important tool for understanding the evolution of COVID-19 lineages and emphasizing the discovery of potent gene-mediated pathways associated with the onset of the disease in humans (6). Based on 346 WGS analysis of SARS-CoV-2, Alteri et al. reported the existence of seven viral lineages causing local transmission, and among them at least two were originated in Italy (7). An evolutionary study revealed a co-expansion tendency of the COVID-19 among neighboring countries of the US with multiple sources and transmission routes for SARS-CoV-2 (8). In January 2021, WHO included B.1.617.2 (Delta variant) as a VOC with spike double mutation E484Q and L452R have led to the second wave in India. Subsequently, in April 2021, Delta has been further substituted to Delta variants such as AY.1, AY.2, and AY.3 contains an additional spike protein substitution K417N.

In India, UP is the most populous state (Census 2011, India) which shares an international transboundary with Nepal. The inter-state migration of the labor/student population around the year is very high due to the lack of industries/educational hubs. The first case of COVID-19 infection in Basti [a district in Eastern Uttar Pradesh (E-UP)] acted as a superspreader event of transmission in the region (9). Eastern Uttar Pradesh experienced a steep rise in COVID-19 cases during the first and second lockdown due to the reverse migration of workers from various states of the country. India has experienced a deadly second wave during the COVID-19 pandemic in 2021 along with many post-vaccination breakthrough infections due to new variants (10). During the second wave of COVID-19, the upsurge of the cases occurred after the Holi festival and travel activities from metro cities from March to April 2021 and deadly variants emerged in the E-UP from various parts of the country (11).

Various studies on the genetic epidemiology of SARS-CoV-2 from India have been carried out (5, 12–14); still, there is a scarcity of genomic data for SARS-CoV-2 from E-UP. The present study was conducted to carry out the molecular surveillance of the SARS-CoV-2 strains, explore clinical association and disease outcomes in the first and second waves of SARS-CoV-2 in E-UP. This will be the first comprehensive study sharing the genetic information of SARS-CoV-2, their distinct lineage/clade cluster, and contribution to the epidemic from this region of Uttar Pradesh, India.

## Materials and Methods

### Sample Acquisition

ICMR-Regional Medical Research Center Gorakhpur (RMRCGKP) is a major testing center for SARS-CoV-2 samples from Gorakhpur and its surrounding districts of the E-UP region of India. During the period of the first wave (March 2020 to February 2021), nasopharyngeal/oropharyngeal (NPS/OPS) (*n* = 339,997) swabs were collected by Integrated Disease Surveillance Project (IDSP) team from districts including Siddharth Nagar, Azamgarh, Basti, Maharajganj, and Deoria, while during the second wave (March 2021 to July 2021) samples (*n* = 360,772) were received for routine COVID-19 diagnosis from Maharajganj and Kushinagar districts through IDSP and a tertiary care medical facility (AIIMS, Gorakhpur) at RMRCGKP. During the period of March 18, 2020 and July 31, 2021, a total of 14,509 samples were tested positive by real-time PCR (RT-PCR). Out of 14,509 samples that gave a positive PCR test, 147 were available for sequencing. The primary inclusion criteria for participants during the study period was SARS-CoV-2 positive samples that exhibited a cycle threshold (Ct) <30 in RT-PCR diagnosis. Hence, a total of 147 positive samples fulfilling the above criteria were randomly selected from April 2, 2020 to July 31, 2021 and subsequently packed in triple layer packing on dry ice according to International Air Transport Association (IATA) protocol and transported to ICMR-NIV, Pune for whole-genome sequencing (WGS). Of these, 87 samples passed quality control and were analyzed further in this study. Samples that had a Ct ≥ 30 and genome recovery of <97% were excluded from the study.

### Demographic Data Extraction and Vaccination Status

The demographic details, clinical history, and associated co-morbidities were extracted in Microsoft Excel from the ICMR COVID-19 data portal (cvstatus.icmr.gov.in). The details regarding vaccination and mortality status were captured by telephonic interview after obtaining verbal informed consent. According to Indian government policy, vaccination was prioritized for all health care workers (HCWs) or citizens more than 45 years of age who can use two doses of BBV152 or ChAdOx1 vaccine at a minimum interval of 4 weeks since January 2021. So that a registration record was obtained regarding first and second doses of vaccination from SARS-CoV-2 positive cases of the second wave. COVID-19 breakthrough infection is defined as the detection of any COVID-19 infection occurring ≥14 days after receiving all recommended doses of either of the vaccines.

### Statistical Analysis

All statistical analyses were performed using IBM SPSS Statistics for Windows, version 20 (IBM Corp., Armonk, NY). Age was expressed using median (interquartile range) and differences of age across two waves were analyzed using Mann-Whitney U-test. All categorical variables including variants were expressed as the frequency with percentage and the differences across two waves were analyzed using either Pearson's Chi-square test or Fischer exact test as applicable. *P*-value < 0.05 was considered to be statistically significant. The case distribution map was generated by using Epi Info 7.2 software program (CDC, Atlanta, GA).

### RNA Extraction and Next Generation Sequencing

Viral nucleic acid was extracted from 200 μl of NPS/OPS using the MagMAX™ Viral pathogen nucleic acid extraction kit as per the manufacturer's instructions (Thermo Fisher Scientific, USA). Library preparation was done using the extracted quantified RNA using TruSeq Stranded mRNA Library Prep Kit. The detailed steps during the library preparation and sample loading are described elsewhere (15, 16). The generated sequence reads were analyzed and mapped using the reference-based assembly method on the CLC Genomics Workbench version 20 (CLC, Qiagen) and used to call mutations in the SARS-CoV-2 genomes. The reference genome, SARS-CoV-2 Wuhan-HU-1 (Accession No.: NC_045512.2) retrieved from the GISAID database (17) was used for mapping.

### Phylogenetic Analysis

Evolutionary analysis was performed with the sequences obtained from this study along with the reference sequences from Uttar Pradesh retrieved from the GISAID database. There were 86 sequences used to generate cladogram with a genome recovery of more than 98.5%. The sequences were aligned using the CLC Genomics Workbench and manually checked for correctness. The nucleotide variations and amino acid substitutions were annotated. A maximum likelihood phylogenetic tree was built using the best substitution model with 1,000 bootstrap replication to assess the statistical robustness using MEGA7 (18).

## Results

### Clinico-Demographic Analysis

**Figure 1:**
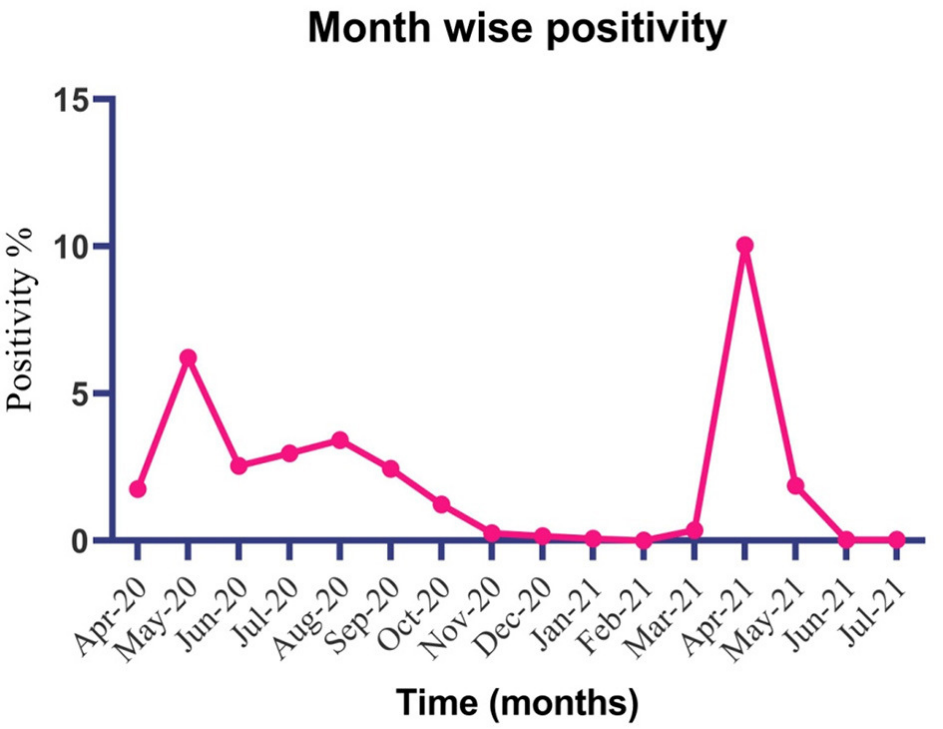
Trend of COVID-19 sample positivity at ICMR-RMRC, Gorakhpur from April-2020 to July 2021.

A total of 700,769 respiratory samples NPS/OPS were tested at ICMR-RMRC, Gorakhpur from March 18, 2020 to July 31, 2021 by SARS-CoV-2 real-time PCR. Among these, a total of 14,509 samples tested positive for SARS-CoV-2. During the period of the first wave (March 2020 to February 2021) of the pandemic, we observed an upsurge of cases in May followed by August, while in the second wave (March 2021 to July 2021) peak cases were witnessed alone in April (Figure 1 and Supplementary Table 1). Furthermore, in the first wave due to lack of a testing facility at adjoining districts of Gorakhpur, we received samples from seven different districts. Whereas, during the second wave after the establishment of testing centers at different district medical care facilities, the samples were limited to two districts only (Maharajganj and Kushinagar) and AIIMS Gorakhpur. The inclusion criteria of the study fulfilling in 147 patients having comparative clinical and demographic details are outlined in Table 1.

**Table 1:** Demographic characteristics, clinical outcomes, and VOCs in the first and second waves.

**Variables**	**March 2020–Feb 2021**	**March 2021–Jul 2021**	***p*** **-Value**
	**First wave (***n*** = 109)**	**Second wave (***n*** = 38)**	
**Age in years [Median (IQR)]**	30 (22–45)	41 (30–56)	0.005^a^
**Gender**			
Female	26 (23.9)	13 (34.2)	
Male	83 (76.1)	25 (65.8)	0.213^b^
**Symptomatic status**	68 (62.4)	30 (79.0)	0.062^b^
**Co-morbid condition**	13 (11.9)	10 (26.3)	0.036^b^
**Death**	1 (0.9)	2 (5.3)	0.328^c^
**Variants of concern (VOC) among genome retrieved**	*n* = 49	*n* = 38	
A.20	1 (2.04)	0	
B.1	14 (28.57)	0	
B.1.1	12 (24.48)	0	
B.1.1.101	1 (2.04)	0	
B.1.1.216	1 (2.04)	0	
B.1.1.306	2 (4.08)	0	
B.1.210	3 (6.12)	0	
B.1.36	6 (12.24)	0	
B.6.6	9 (18.36)	0	
B.1.617.1	0	3 (7.9)	
B.1.617.2	0	27 (71.0)	
AY.1	0	8 (21.1)	

a *Mann-Whitney U-test*.

b *Pearson's Chi-square test*.

c *Fischer exact test*.

The clinico-demographic features of the 147 samples subjected to WGS have been reflected in two groups as the first wave (*n* = 109) and the second wave (*n* = 38) in Table 1. Of the 109 individuals in the first wave, the median age of the individuals in the study was 30 years (IQR 22–45) and males predominated (83; 76.1%). Of the 68 (62.3%) symptomatic individuals, the most common symptoms reported were cough (60/68; 88.2%), fever (56/68; 82.35%), and headache (25/68; 36.76%) followed by shortness of breath (6/68; 8.8%), sore throat (6/68; 8.8%), loss of taste (3/68; 4.4%), and diarrhea (1/68; 1.47%). At least one of the co-morbid conditions was noted in 13 (11.9%) individuals, among whom diabetes mellitus (10/13; 76.9%) was most commonly reported followed by hypertension (5/13, 38.46%) and chronic lymphocytic leukemia (1/13, 7.6%). Of the 109 individuals, only one succumbed to death having associated leukemia. Vaccination details were available for 49 individuals used for WGS, all were unvaccinated.

Of the 38 individuals in the second wave, the median age was 41 years (IQR 30–55.75). Most of them were males (25; 65.8%) and symptomatic (30; 78.9%). Among the symptomatic individuals, the most common symptoms were fever (29/30; 96.6%), cough (25/30; 83.3%), loss of smell (11/30; 36.6%), shortness of breath (7/30; 23.3%), sore throat (4/30; 13.3%), headache (2/30; 6.6%), and diarrhea (1/30; 3.3%). Co-morbid conditions were noted in 10 (26.3%) individuals, of whom diabetes mellitus (7/10; 70%) was the most followed by hypertension (4/10, 40%). Twenty-four of them were unvaccinated. Two individuals succumbed to death and both were unvaccinated. A total of 14 individuals were vaccinated, among whom COVISHIELD (ChAdOx1) and COVAXIN (BBV152) were received in 11 and 3 individuals, respectively. Among these five tested positive with a median time between the day of testing positive and at least first dose is 41 days (IQR 29–70). Of these five individuals, only two had received a second dose; the median time between the receipt of the second dose and testing positive was 61.5 days. A statistically significant (*p*-value < 0.005) difference in age group affected and association of co-morbid condition was noted in first and second waves.

### Whole-Genome Sequence Analysis

Of the 147 cases subjected to complete genome sequencing, 97–100% coverage was obtained for 87 cases. Of these 87 samples, 49 were from the first wave while the rest of the 38 sequences obtained were from the samples recovered during the second wave. The observed average (Ct) value for these cases was 22.4.

**Figure 2:**
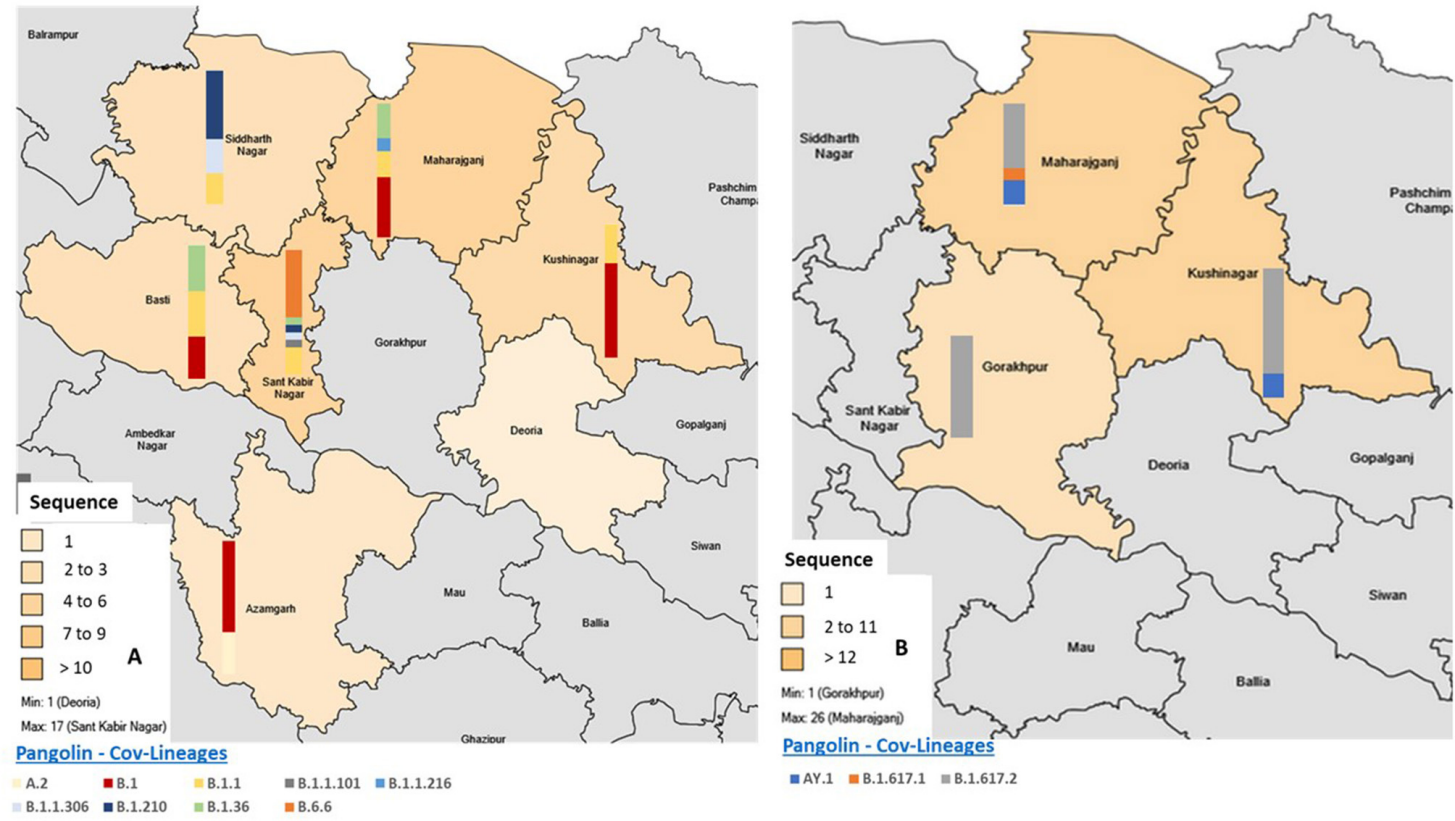
Geographic distribution of COVID-19 patients from which SARS-CoV-2 genomes were sequenced and breakdown of sequenced cases according to the pangolin lineages across Eastern Uttar Pradesh. **(A)** Sequencing and diversity of SARS-CoV-2 sequences obtained in the first wave and **(B)** sequencing and diversity of SARS-CoV-2 sequences obtained in the second wave in Eastern Uttar Pradesh. The y-axis of bars is given in the Supplementary Figures 1A,B.

In the first wave the majority of the cases showed similarity with SARS-CoV-2 GR clade (40.8%), followed by unclassified clade O (24.4%). The other samples comprised 22.4% of GH, 10.2% of G, and the least samples showed similarity with the S clade (2.04%). Whereas, during the second wave only G clade (100%) was found, no other clade was detected. Further, according to the pangolin lineage the samples from the second wave of the pandemic, all VOCs responsible for case fatalities were detected with a majority of Delta variant (B.1.617.2) (71.1%), followed by the Delta plus (AY.1) in 21.1%, and the Kappa variant (B.1.617.1) in 7.9%. The distribution of the cases and lineages/variants in the first wave (*n* = 49) and in the second wave (*n* = 38) from eight districts of E-UP is provided in Figure 2.

A total of 87 SARS-CoV-2 genome sequences were retrieved with a genome coverage of more than 98.5%. The details of the percent genome coverage, total reads, and relevant reads are given in Supplementary Table 2. The pangolin lineage of the above sequences was also obtained using the web version of PANGOLIN software (https://Pangolin.cog-uk.io/). The Pangolin lineages for each sequence are given in Supplementary Table 2. Genome analysis of the retrieved 87 SARS-CoV-2 genomic sequences demonstrated 135 amino acid (aa) variation across the genome when compared to a reference. A list of aa substitutions observed in the spike protein is mentioned in Table 2. Earlier GISAID described variations that were observed during the first wave. It is noteworthy that substitution D614G in spike (S) protein was found in all G and its variant clade GH and GR, while Q57H substitution located in ORF3a protein was only found in 15 cases distributed among GH (*n* = 9), GR (*n* = 5), and O (*n* = 1) clade from the first wave (Supplementary Table 3).

**Table 2:** Residue substitution in spike protein among various lineages of SARS-CoV-2 virus.

**Pangolin lineage**	**Amino acid substitutions in spike region compared with Wuhan-Hu-1 isolate**
A.20	ND*
B.1	L18R, L24S, S477N, F490S, D574Y, E583D, D614G
B.1.1	L18R, D614G
B.1.1.101	D614G
B.1.1.216	D614G
B.1.1.306	D614G
B.1.210	L18R, L24S, F490S, A522V, D614G
B.1.36	L18R, T95S, K558N, L585H, D614G
B.1.617.1	T95I, E154K, L452R, E484Q, D614G, P681R
B.1.617.2	T19R, T95I, A222V, G446V, L452R, T478K, D574Y, D614G, P681R
AY.1	T19R, T95I, W258L, K417N, L452R, T478K, D614G, P681R
B.6.6	ND*

* *ND, not detected*.

### Comparison of Key Residues in SARS-CoV-2 Variants

**Figure 3:**
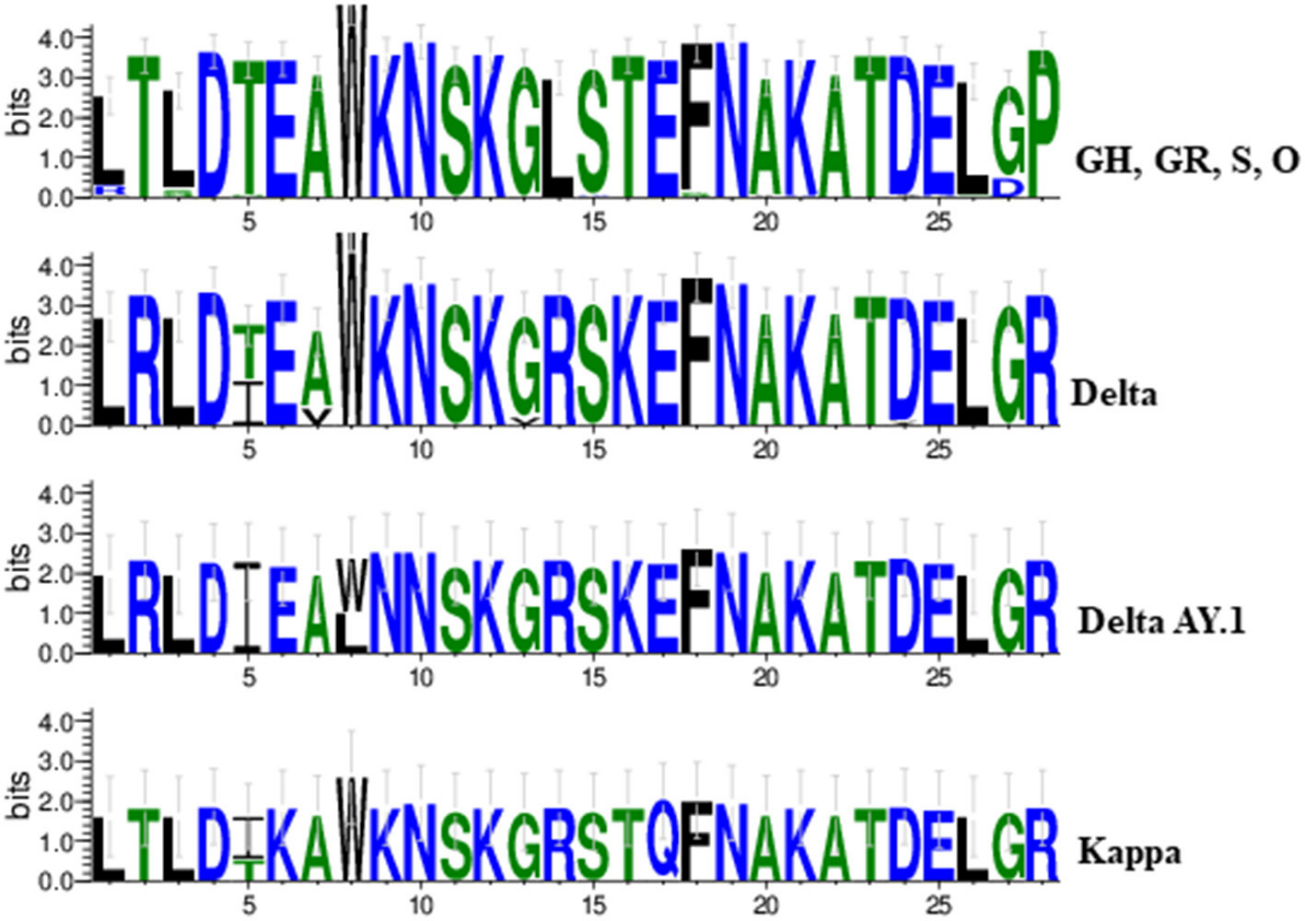
A sequence logo representation of the SARS-CoV-2 spike protein in which letter height reflects the likelihood of finding a particular residue in that position. Residues are colored according to hydrophobicity (green-hydrophobic, blue-hydrophilic).

Overall variations at 21 aa positions were found at receptor binding domain (RBD) of the spike protein throughout the first and second wave cases. Signature substitutions at positions L452R and P681R were commonly detected in the Delta, Delta AY.1, and Kappa variant whereas T19R, T478K, and D614G were confined to Delta and Delta AY.1 variant only. Further, an exceptional mutation was present at A222V among Delta, and K417N among Delta AY.1 variants. In the second wave of 38 cases, aa variation at T95I was detected in 71.4% cases (Delta = 12, Delta AY.1 = 6, and Kappa = 2). However, the Kappa variants demonstrated signature substitution at E154K and E484Q. The uncommon aa change D574Y was uniquely detected in two variants including GR clade (MCL-20-H-405) and Delta variant (MCL-21-6602). Additionally, in the spike region of Delta AY.1 variant unique substitution at W258L was detected in 37.5% cases (n=3/8). We also identified 8 substitution mutants in the SARS-CoV-2, K417N, G446V, L452R, S477N, T478K, E484Q, F490S, and A522V in the RNA binding domain (RBD) of S1 subunit of Spike protein. Two mutations in Spike (K417N and W258L) were exclusively present in the Delta Plus variant. Interestingly, all three Kappa variants had two unique mutations (T1567I and M5753I) in ORF1ab protein. Further, the impact of variation in the spike protein among SARS-CoV-2 variants we compared the sequence conservation among Delta, Delta AY.1, Kappa, and other lineages obtained in this study. Tryptophan was the most common residue (W258, 87/90) in Delta and Kappa variant (Figure 3).

### Phylogenetic Analysis

**Figure 4:**
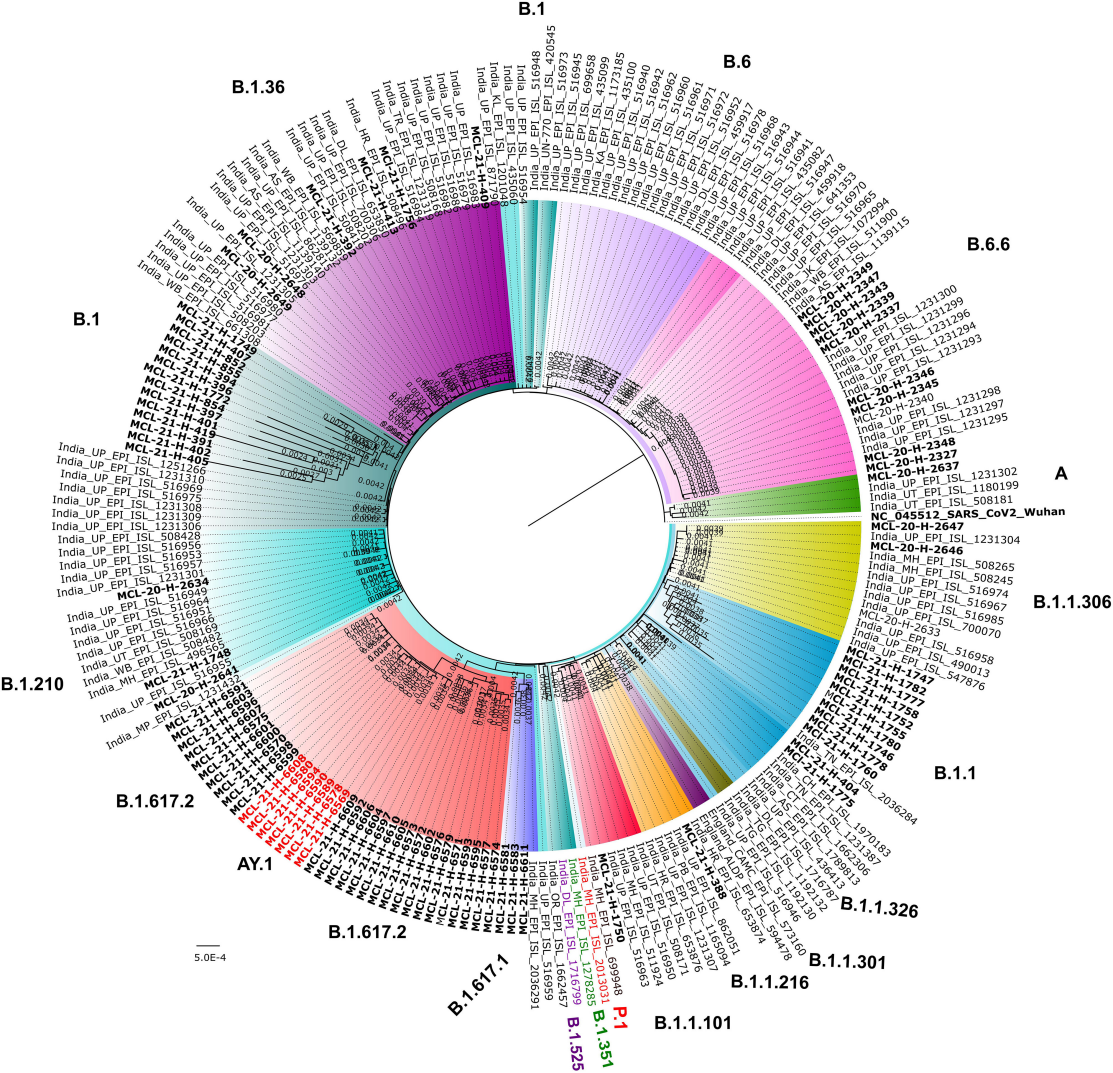
Maximum likelihood tree of the SARS-CoV-2 sequences. A maximum likelihood tree was built for the sequences retrieved in our study along with other GISAID sequences from Uttar Pradesh, India using the best substitution model. A bootstrap replication of 1,000 cycles was performed to assess the statistical robustness. SARS-CoV-2 sequences retrieved in the study are marked in bold black color. The nodes and branches are marked in different colors.

The evolutionary analysis revealed the circulation of GR, O, GH, G, and S clade during the first wave of the pandemic whereas only G clade variants were detected as the predominant strain during the second wave of infection. Among the studied cases, one sequence (MCL-20-H-2637) demonstrated genetic relatedness to the Wuhan isolate. The Pangolin lineage B.1.617.1 (Kappa) from this study showed the closest match with the sequence retrieved from Maharashtra (India_MH_EPI_ISL_2036291). The Pangolin lineage B.1.617.2 (Delta) and AY.1 (Delta plus) were detected for the first time from the E-UP region (Figure 4).

### Impact of Vaccination on SARS-CoV-2 Infections

In the second wave of 38 cases, five patients (three from Maharajganj, one from Kushinagar, and one from Gorakhpur) were identified as breakthrough infections. The clinical samples for the analysis were collected between April–May, 2021. Out of these five patients, three acquired COVID-19 infection after taking the first dose of the vaccine, while two were infected after receiving both doses of the vaccine. A total of three patients had received ChAdOx1 vaccine and two had received BBV152 vaccine.

**Figure 5:**
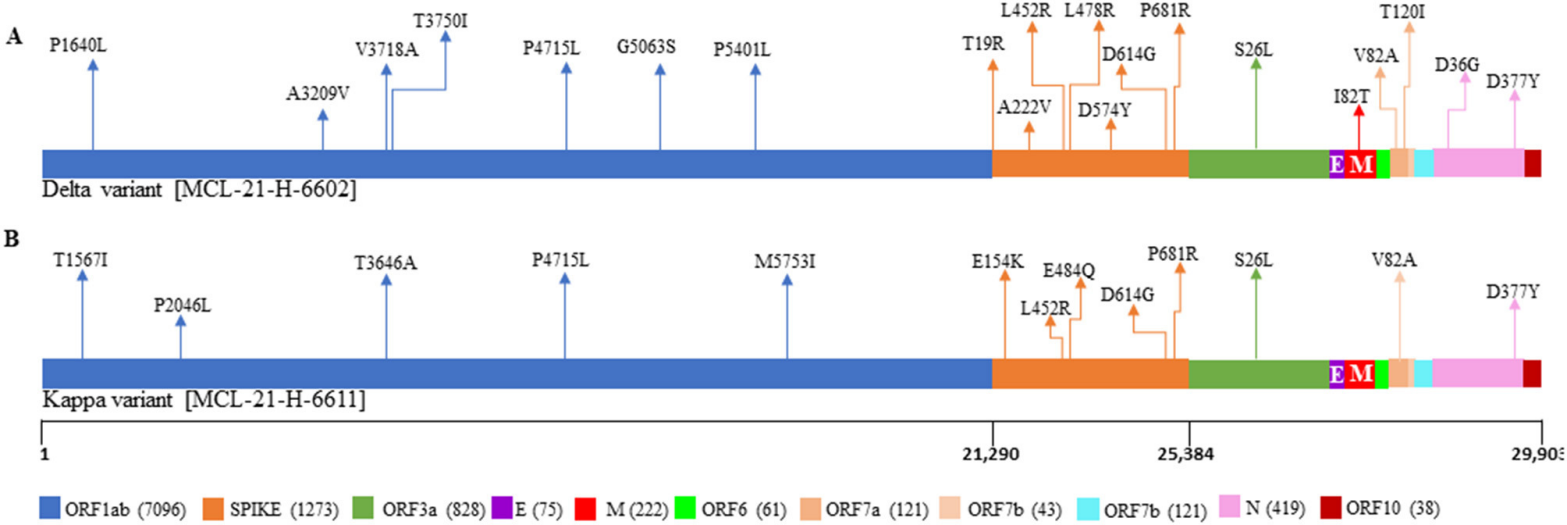
Representation of mutations within SARS-CoV-2 genome for the patients with the vaccine breakthrough infections. The plot displays the different locations of the mutations observed in each sample, with different colors corresponding to different genes. **(A)** Represents the mutations from the patient breakthrough sequence Delta variant. **(B)** Represents the mutations from the breakthrough sequence Kappa variant.

Clinical data were analyzed for five breakthrough cases. The median age (and the IQR) of patients in the study was 50 (30–63), with the breakthrough cases after one dose were 50 (45–56) and after two doses were 46.5 (30 and 63) (Supplementary Table 2). A total of 3 (60%) of the breakthrough cases were males. Delta plus [AY.1] (*n* = 3) was the major SARS-CoV-2 lineage observed in the individuals who received only the first dose of the vaccine. Notably, of the two breakthrough infection cases, the first was due to the Delta (B.1.617.2) and the second breakthrough infection was due to the Kappa (B.1.617.1) variant. Both the variants belong to clade G. Mutation analysis revealed that the samples from the case of Delta variant breakthrough infection contained 20 non-synonymous mutations and the case of the Kappa variant contained 13 non-synonymous substitutions (Figure 5). A total of seven shared mutations were found between Delta and Kappa breakthrough infection cases. Interestingly, an exceptional aa change was detected at position D574Y in two cases, one from GR clade and one from Delta variant with breakthrough infection, whereas another breakthrough infection from the Kappa variant conserved the aa change at position 95 (T) as in the Wuhan isolate.

## Discussion

In India, an alert for the air-borne virus transmission was created, since the identification of the first case of SARS-CoV-2 from the Kerala state (2). Following this, the Ministry of Home Affairs (MHA) announced the country-wide lockdown (March 25, 2020 to April 14, 2020) to control and prevent the spread of viral transmission. The previous WGS analysis of SARS-CoV-2 cases introduced to India from other countries demonstrated remarkable genetic diversity (19). The rural areas of the E-UP region accommodate primarily the migratory population/laborers and frequent travel for job or education was witnessed from this region. Hence, it is likely that the virus was introduced to this region through migratory movements of the population. Our previous study demonstrated that during the first wave of the pandemic, a student migrated to Sant Kabir Nagar from Deoband and spread the infection to the familial cluster (20).

Epidemiological analysis of SARS-COV-2 clinical samples obtained during the period of the first wave determined that GH and GR clade were the prevalent strains in different Indian states (5). The predominance of the GR clade was also confirmed by the WGS analysis derived from the first wave of this study, suggesting that the circulation of this clade was mostly reported from the southern part of India. However, the unclassified cluster followed the majority of the cases after the GR clade from the E-UP region.

Like different states of India, E-UP has experienced two massive COVID-19 spikes with a significant case of fatality rates, alarming an urgent need for its effective treatment based on antiviral medicine and vaccines that reduce the mortality and morbidity rates due to COVID-19. The major affected states were Maharashtra, Kerala, Karnataka, Andhra Pradesh, Tamil Nadu, Delhi, Uttar Pradesh, and West Bengal. Despite the high caseload during the second wave, several national movements such as the election in several states, political rallies, farmer protests, and mass gatherings in religious places were going on in an uncontrolled manner that leads to the spread of COVID-19 beyond the urban center to rural regions (11). During the second wave of the pandemic, VOCs such as Delta and Delta AY.1 were introduced in the E-UP region. An upsurge of SARS-CoV-2 cases was observed at the end of March 2021 in this region. A trend toward rising cases was seen and local spread of SARS-CoV-2 in this region was observed during April 2021. Recently, a study identified 56 distinctive single nucleotide polymorphism (SNP) variations among SARS-CoV-2 in central UP that are majorly clustered into two groups which shows the deleterious effects on the genome (21).

Based on genetic similarity and full-length SARS-CoV-2 genome sequences, most cases diagnosed during the early phase of the first wave of SARS-CoV-2 infection (April to May, 2020) in the E-UP region appear to be associated with exposures or contacts of travelers visiting this region after implementation of the lockdown to curtail the SARS-CoV-2 introductions. The majority of early introductions in April and May 2020 appear to have been sourced from Southeast Asia (B.6.6), Europe (B.1), and other parts of India (B.1.210 and B.1.247) (5). Maximum genetic diversity (in terms of Pangolin lineages) among the circulating SARS-CoV-2 viruses was observed in the course of the first wave in this part of India. Several new SARS-CoV-2 variants of concern and interest (VoC/VoI), i.e., Alpha (B.1.1.7), Beta (B.1.351), and Gamma (B.1.1.28.1), had been detected from various parts of India during the first wave of COVID-19 (5, 22). However, this study demonstrates the absence of the VoC/VoI in this region during the first wave.

During the second wave, Kappa (B.1.617), Delta (B.1.617.2), and Delta (AY.1) emerged as the major sub-lineages from India (23). In the second wave of the pandemic, the E-UP was affected with Kappa, Delta, and Delta variants. As of July 2021, a total of 959 SARS-CoV-2 genome data available from Uttar Pradesh, India at INSACOG revealed the diversification and clustering of seven pangolin lineages (http://clingen.igib.res.in/covid19genomes/). These were followed by unclassified clustered sequences which lie in B.6, B.6.1, and B.6.6 pangolin lineage. The SARS-CoV-2 sequences retrieved from migrants also fall in B.1.210, B.1.306, and B.1.36. This indicates that despite the earlier classification group of G, GR, and GH clades, these have a distinct signature as per current pangolin nomenclature. The second wave of the SARS-CoV-2 had a majority of the Delta and Delta AY.1 variants of the virus spread through local transmission. Interestingly, cases during the second wave did not have any SARS-CoV-2 variants similar to the first wave except for G clade which was accounted for 9.6%, which revealed unique patterns of transmission.

The Spike protein of coronavirus is crucial for viral entry into the host cells and pathogenesis with the most variable sequences. The receptor-binding domain (RBD) has the most genomic variation in SARS-CoV-2 spike glycoprotein (24). The residues found in RBD might be crucial for SARS-CoV-2 binding to hACE2 which provides clues for monitoring the increased infectibility of natural RBD mutations during the transmission of the virus (25). SARS-CoV-2 spike protein substitution D614G variant is suggested to confer higher viral infectivity, efficient replication, mortality rate, and immune system evasion and transmission (26–28). The breakthrough infections showed aa changes at position S:D574Y in the cases of Delta variant, whereas from Kappa variant conserved residue at position 95 (T) suggesting that these variations are unlikely to reduce the ability to protect against COVID-19 infection.

During the second wave of COVID-19, the emergence of new SARS-CoV-2 lineage B.1.617 and sublineage B.1.617.2 (Delta variant), a “variant of concern” in India, has been associated with a surge in daily infections along with some breakthrough infections (22). The Delta variants are more than twice as transmissible as the original strain of SARS-CoV-2 (29, 30) and are capable of escaping the host immune response (31), which leads to breakthrough infection. Our study also depicts the distinct wave of two massive COVID-19 spikes in the E-UP region, accompanied by a high number of positive cases in the second wave along with 2 (5.26%) cases of breakthrough infection. Older age factors delay the development of immunity may contribute to increased susceptibility to VOCs. Recently, Beta and Delta variants have been reported to partially evade immunity after recommended doses of vaccination. It is known that many anti-RBD-specific antibodies can only bind to the open spike protein. Plausibly, mutations altering the conformation of the spike glycoprotein make the RBD less susceptible to neutralizing antibodies (32–34).

## Conclusion

In summary, two waves of the COVID-19 pandemic were documented in the E-UP. The first wave mostly included patients who had returned from interstate and the second one was local spread possibly due to mass gatherings. Dynamics of SARS-CoV-2 circulating in the E-UP region were dominated by SARS-CoV-2 variants belonging to clades GH, GR, G, S, and O from the first wave. In contrast, the cases recorded in the second wave were strongly predominated by G clade variant, Delta and its variants, and Kappa. The signature amino acid changes were identified for the pandemic deadly variants. The breakthrough infections showed unique mutational changes at position S:D574Y in cases of the Delta variant, whereas from the Kappa variant conserved aa change at position 95 (T) as in the Wuhan isolate.

## Data Availability Statement

The datasets presented in this study can be found in online repositories. The names of the repository/repositories and accession number(s) can be found in the article/Supplementary Material.

## Ethics Statement

The ethical clearance was approved by the Institutional Ethics Committee (ICMR-RMRC, Gorakhpur) with IHEC ID No. RMRCGKP/EC/2020/2.2. Written informed consent from the participants' legal guardian/next of kin was not required to participate in this study in accordance with the national legislation and the institutional requirements.

## Author Contributions

HD, PY, SM, and SB: contributed to conceptualization and investigation. DN, AS, PP, AKu, and SM: methodology and software. BM, SB, SM, and PB: formal analysis. KZ: clinical interpretation. HD, KZ, SB, SM, and PB: writing original draft preparation. GD, RS, BM, AP, NK, GY, MK, SG, AKa, RSS, and SP: resources. RK, HD, and PY: supervision, review, and editing. All authors read and approved the final manuscript.

## Funding

This work was supported by the Indian Council of Medical Research (ICMR), New Delhi provided intramural funding to ICMR-RMRC, Gorakhpur and ICMR-National Institute of Virology, Pune.

## Conflict of Interest

The authors declare that the research was conducted in the absence of any commercial or financial relationships that could be construed as a potential conflict of interest.

## Publisher's Note

All claims expressed in this article are solely those of the authors and do not necessarily represent those of their affiliated organizations, or those of the publisher, the editors and the reviewers. Any product that may be evaluated in this article, or claim that may be made by its manufacturer, is not guaranteed or endorsed by the publisher.

## References

[1] Zhou P Yang XL Wang XG Hu B Zhang L Zhang W. A pneumonia outbreak associated with a new coronavirus of probable bat origin. Nature. (2020) 579:270–3. 10.1038/s41586-020-2012-7 32015507 PMC7095418

[2] Andrews MA Areekal B Rajesh KR Krishnan J Suryakala R Krishnan B. First confirmed case of COVID-19 infection in India: a case report. Indian J Med Res. (2020) 151:490–2. 10.4103/ijmr.IJMR_2131_20 32611918 PMC7530459

[3] WHO Coronavirus Disease (COVID-19) Dashboard. Available online at: https://covid19.who.int/ (accessed November 12, 2021).

[4] Rambaut A Holmes EC O'Toole Á Hill V McCrone JT Ruis C. Addendum: a dynamic nomenclature proposal for SARS-CoV-2 lineages to assist genomic epidemiology. Nat Microbiol. (2021) 6:415. 10.1038/s41564-021-00872-5 33514928 PMC7845574

[5] Yadav PD Nyayanit DA Majumdar T Patil S Kaur H Gupta N. An epidemiological analysis of SARS-CoV-2 genomic sequences from different regions of India. Viruses. (2021) 13:925. 10.3390/v13050925 34067745 PMC8156686

[6] Schultze JL Aschenbrenner AC. COVID-19 and the human innate immune system. Cell. (2021) 184:1671–92. 10.1016/j.cell.2021.02.029 33743212 PMC7885626

[7] Alteri C Cento V Piralla A Costabile V Tallarita M Colagrossi L. Genomic epidemiology of SARS-CoV-2 reveals multiple lineages and early spread of SARS-CoV-2 infections in Lombardy, Italy. Nat Commun. (2021) 12:434. 10.1038/s41467-020-20688-x 33469026 PMC7815831

[8] Pan B Ji Z Sakkiah S Guo W Liu J Patterson TA. Identification of epidemiological traits by analysis of SARS-CoV-2 sequences. Viruses. (2021) 13:764. 10.3390/v13050764 33925388 PMC8145049

[9] Kant R Zaman K Shankar P Yadav R Team RGCD. A preliminary study on contact tracing and transmission chain in a cluster of 17 cases of severe acute respiratory syndrome coronavirus 2 infection in Basti, Uttar Pradesh, India. Indian J Med Res. (2020) 152:95–9. 10.4103/ijmr.IJMR_2914_20 32811800 PMC7853285

[10] Gupta N Kaur H Yadav P Mukhopadhyay L Sahay RR Kumar A. Clinical characterization and Genomic analysis of COVID-19 breakthrough infections during second wave in different states of India. medRxiv. (2021) 2021.2007.2013.21260273. 10.1101/2021.07.13.21260273 PMC8472862 34578363

[11] Kar SK Ransing R Arafat SMY Menon V. Second wave of COVID-19 pandemic in India: barriers to effective governmental response. EClinicalMedicine. (2021) 36:100915. 10.1016/j.eclinm.2021.100915 34095794 PMC8164526

[12] Abraham P Cherian S Potdar V. Genetic characterization of SARS-CoV-2 and implications for epidemiology, diagnostics and vaccines in India. Indian J Med Res. (2020) 152:12–5. 10.4103/ijmr.IJMR_3667_20 32896836 PMC7853292

[13] Banu S Jolly B Mukherjee P Singh P Khan S Zaveri L. A distinct phylogenetic cluster of Indian severe acute respiratory syndrome coronavirus 2 isolates. Open Forum Infect Dis. (2020) 7:ofaa434. 10.1093/ofid/ofaa434 33200080 PMC7543508

[14] Radhakrishnan C Divakar MK Jain A Viswanathan P Bhoyar RC Jolly B. Initial insights into the genetic epidemiology of SARS-CoV-2 isolates from Kerala suggest local spread from limited introductions. Front Genet. (2021) 12:630542. 10.3389/fgene.2021.630542 33815467 PMC8010186

[15] Yadav PD Potdar VA Choudhary ML Nyayanit DA Agrawal M Jadhav SM. Full-genome sequences of the first two SARS-CoV-2 viruses from India. Indian J Med Res. (2020) 151:200–9. 10.4103/ijmr.IJMR_663_20 32242873 PMC7258756

[16] Yadav PD Whitmer SLM Sarkale P Fei Fan Ng T Goldsmith CS Nyayanit DA. Characterization of novel reoviruses wad medani virus (Orbivirus) and kundal virus (coltivirus) collected from hyalomma anatolicum ticks in india during surveillance for Crimean Congo hemorrhagic fever. J Virol. (2019) 93:e00106–19. 10.1128/JVI.00106-19 30971476 PMC6580951

[17] Shu Y McCauley J. GISAID: global initiative on sharing all influenza data - from vision to reality. Euro Surveill. (2017) 22:30494. 10.2807/1560-7917.ES.2017.22.13.30494 28382917 PMC5388101

[18] Kumar S Stecher G Tamura K. MEGA7: molecular evolutionary genetics analysis version 7.0 for bigger datasets. Mol Biol Evol. (2016) 33:1870–4. 10.1093/molbev/msw054 27004904 PMC8210823

[19] Muttineni R Kammili N Bingi TC Rao MR Putty K Dholaniya PS. Clinical and whole genome characterization of SARS-CoV-2 in India. PLoS ONE. (2021) 16:e0246173. 10.1371/journal.pone.0246173 33529260 PMC7853523

[20] Zaman K Shankar P Yadav PD Nyayanit DA Behera SP Bhardwaj P. Molecular epidemiology of a familial cluster of SARS-CoV-2 infection during lockdown period in Sant Kabir Nagar, Uttar Pradesh, India. Epidemiol Infect. (2021) 149:e200. 10.1017/S095026882100198930886898

[21] Prasad P Prakash S Sahu K Singh B Shukla S Mishra H. Unique mutational changes in SARS-CoV2 genome of different state of India. bioRxiv. (2020) 2020.2008.2024.265827. 10.1101/2020.08.24.265827

[22] Thangaraj JWV Yadav P Kumar CG Shete A Nyayanit DA Rani DS. Predominance of delta variant among the COVID-19 vaccinated and unvaccinated individuals, India, May 2021. J Infect. (2021). 10.1016/j.jinf.2021.08.006. [Epub ahead of print]. 34364949 PMC8343391

[23] Kannan SR Spratt AN Cohen AR Naqvi SH Chand HS Quinn TP. Evolutionary analysis of the Delta and Delta Plus variants of the SARS-CoV-2 viruses. J Autoimmun. (2021) 124:102715. 10.1016/j.jaut.2021.102715 34399188 PMC8354793

[24] Wu F Zhao S Yu B Chen YM Wang W Song ZG. A new coronavirus associated with human respiratory disease in China. Nature. (2020) 579:265–9. 10.1038/s41586-020-2008-3 32015508 PMC7094943

[25] Shang J Ye G Shi K Wan Y Luo C Aihara H. Structural basis of receptor recognition by SARS-CoV-2. Nature. (2020) 581:221–4. 10.1038/s41586-020-2179-y 32225175 PMC7328981

[26] Hou YJ Chiba S Halfmann P Ehre C Kuroda M Dinnon KH III 3rd. SARS-CoV-2 D614G variant exhibits efficient replication *ex vivo* and transmission *in vivo*. Science. (2020) 370:1464–8. 10.1126/science.abe8499 33184236 PMC7775736

[27] Volz E Hill V McCrone JT Price A Jorgensen D O'Toole A. Evaluating the effects of SARS-CoV-2 spike mutation D614G on transmissibility and pathogenicity. Cell. (2021) 184:64.e11–75.e11. 10.1101/2020.07.31.20166082 33275900 PMC7674007

[28] Kim SJ Nguyen VG Park YH Park BK Chung HC. A novel synonymous mutation of SARS-CoV-2: is this possible to affect their antigenicity and immunogenicity? Vaccines (Basel). (2020) 8:220. 10.3390/vaccines8020220 32422894 PMC7349911

[29] Cherian S Potdar V Jadhav S Yadav P Gupta N Das M. SARS-CoV-2 spike mutations, L452R, T478K, E484Q and P681R, in the second wave of COVID-19 in Maharashtra, India. Microorganisms. (2021) 9:1572. 10.3390/microorganisms9071542 34361977 PMC8307577

[30] Li B Deng A Li K Hu Y Li Z Xiong Q. Viral infection and transmission in a large, well-traced outbreak caused by the SARS-CoV-2 Delta variant. medRxiv. (2021) 2021.2007.2007.21260122. 10.1101/2021.07.07.21260122 PMC8786931 35075154

[31] Planas D Veyer D Baidaliuk A Staropoli I Guivel-Benhassine F Rajah MM. Reduced sensitivity of SARS-CoV-2 variant Delta to antibody neutralization. Nature. (2021) 596:276–80. 10.1038/s41586-021-03777-9 34237773

[32] Barnes CO Jette CA Abernathy ME Dam KA Esswein SR Gristick HB. SARS-CoV-2 neutralizing antibody structures inform therapeutic strategies. Nature. (2020) 588:682–7. 10.1038/s41586-020-2852-1 33045718 PMC8092461

[33] Shastri J Parikh S Aggarwal V Agrawal S Chatterjee N Shah R. Severe SARS-CoV-2 breakthrough reinfection with delta variant after recovery from breakthrough infection by alpha variant in a fully vaccinated health worker. Front Med (Lausanne). (2021) 8:737007. 10.3389/fmed.2021.737007 34490316 PMC8418387

[34] Weissman D Alameh MG de Silva T Collini P Hornsby H Brown R. D614G spike mutation increases SARS CoV-2 susceptibility to neutralization. Cell Host Microbe. (2021) 29:23.e24.−31.e24. 10.1016/j.chom.2020.11.012 33306985 PMC7707640

