# Supplementary figures and images for: Genome Sequencing Reveals a Mixed Picture of SARS-CoV-2 Variant of Concern Circulation in Eastern Uttar Pradesh, India
Source: Front Med (Lausanne). 2022 Jan 7;8:781287. doi: 10.3389/fmed.2021.781287 (PMC8777020; doi:10.3389/fmed.2021.781287)

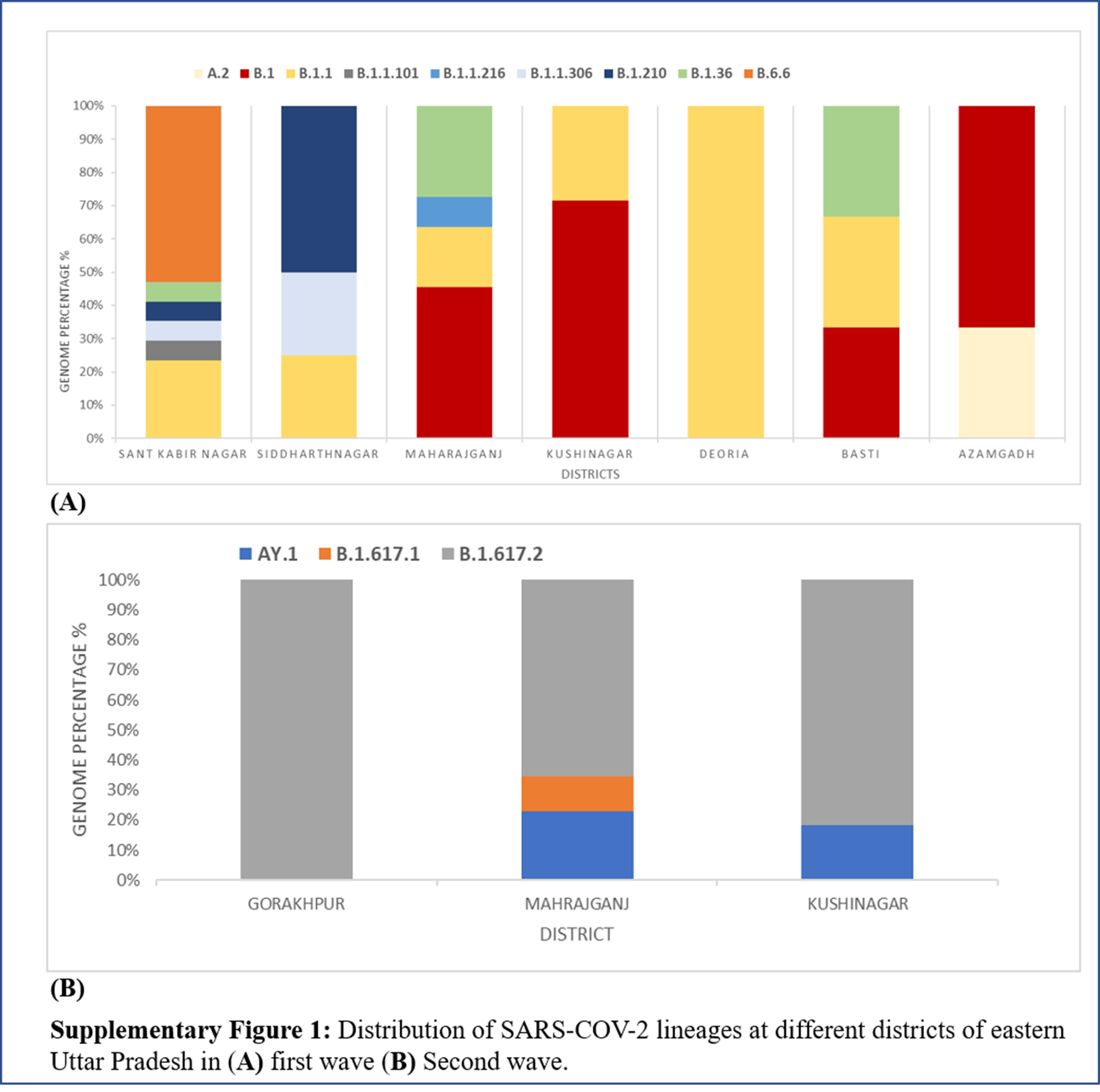

Supplement: Supplementary file 4 [file Image_1.TIF]
